# Supplementary material for: mTOR dysregulation induces IL-6 and paracrine AT2 cell senescence impeding lung repair in lymphangioleiomyomatosis
Source: Nat Commun. 2025 Oct 9;16:8996. doi: 10.1038/s41467-025-64036-3 (PMC12511327; doi:10.1038/s41467-025-64036-3)

mTOR dysregulation induces IL6 and paracrine AT2 cell senescence impeding lung repair in lymphangioleiomyomatosis.

Roya Babaei-Jadidi, Debbie Clements, Yixin Wu, Ken Chen, Suzanne Miller, Manuela Platé, Kyungtae Lim, Ryan Rue, Vera P Krymskaya, Rachel C Chambers, Emma Rawlins, Yan Xu, Simon R Johnson.

Supplementary methods, tables and figures

Supplementary methods.

Patient samples.

Serum samples and lung tissue were obtained from patients receiving care at the UK LAM Centre between 2011 and 2022. All patients had LAM as defined by American Thoracic Society / Japanese Respiratory Society criteria[1]. Tissue samples were taken for clinical care, . Linked clinical data was captured from medical notes including lung function (FEV_1, DL_{CO} and six-minute walk distance) and VEGF-D were recorded at baseline and lung function repeated at subsequent visits. Change in lung function was calculated as the slope of a regression line of all values of FEV_1 (ΔFEV_1) or DL_{CO} (ΔDL_{CO}). Clinical characteristics of patient samples used for laser capture microdissection and immunohistochemistry are shown in Supplementary table 1. The study was approved by the East Midlands Research Ethics Committee (reference 13/EM/0264) and all participants gave written informed consent.

Cell, LAM spheroid and alveolar organoid culture

TSC2-null 621-101 and *TSC2*–add-back 621-103 cells were a gift from Dr. Lisa Henske (Brigham and Women's Hospital). Cells were cultured in DMEM/F-12, no phenol red (Fisher scientific, 11580546) with FBS[2]. TTJ cells derived from renal tumours of *TSC2*$^{+/-}$ C57BL/6 mice, were provided by Dr. Vera Krymskaya (University of Pennsylvania). Primary LAM associated fibroblasts were obtained from lung tissue from women with LAM and LAM spheroids comprising 621 cells and LAFs were prepared from 10,000 cells in ultra-low attachment 96-well plates as previously described[3]. For coculture 621-101 or 621-103 cells seeded into each well of a Boyden chamber (Transwell, Corning) and fibroblast were seeded onto the insert. Human foetal lung tissue was provided from terminations of pregnancy from the MRC/Wellcome Trust Human Developmental Biology Resource (London and Newcastle, University College London (UCL) site REC reference: 18/LO/0822; Project 200591; www.hdbr.org)[4]. Sample age ranged from 17-22 weeks of gestation and had no known genetic abnormalities. Sample gender was unknown at the time of collection and was not determined. AT2 cells were derived and cultured in organoids as described[5 6]. Cryopreserved organoids were warmed in a water bath at 37°C for 2

minutes, transferred to a 15 mL Falcon conical tube in 9 mL of fresh basal medium, centrifuged at 500 g for 5 min, the supernatant removed and the pellet resuspend in 25 µL Matrigel which was incubated at 37°C for 15 minutes prior to adding AT2 medium[5].

Laser capture microdissection

Tissue sections were mounted on PEN Membrane Glass Slides (Thermo Fisher scientific LCM0522), deparaffinised in xylene for 2 minutes, twice, 100% ethanol for 1 minute, 96% ethanol for 1 minute then 70% ethanol for 1 minute and stained using Cresyl Violet. LCM was carried out using a Zeiss AxioImager Z1 (Zeiss, Thornwood, NY), using αSMA and PNL2 staining to identify LAM nodules. Microdissected samples were captured by Adhesive Cap 500ul microcentrifuge tubes (Fisher Scientific, Carl Zeiss 415190-9211-000). RNA extraction was performed by using
RNeasy DSP FFPE Kit (QIAGEN, ID: 73604). Genomic DNA was eliminated using, RQ1 RNase-Free DNase (Promega, M6101) and clean-up performed using Monarch RNA Cleanup Kit (Biolabs, New England, T2030L).

Library Preparation and RNA Sequencing

Laser captured RNA samples were quantified using Ribogreen QuanT-iT kit on a Fluostar Plate reader and quality checked using a high sensitivity RNA Tape on a 4200 TapeStation. The RIN number was between 1.7 to 3.3. and the percentage of RNA molecules that are greater than 200bp in length (DV200 score) also calculated, with samples with higher than 35% DV200 chosen. The library preparation and RNA sequencing was performed by Birmingham Genomic centre using Lexogen QuantSeq 3' mRNA-Seq Library Prep Kit FWD from Illumina. Libraries were quantified using a PicoGreen Quant-iT kit and sized with a D1000 tape. For sequencing, libraries were pooled in equal volumes according to their molarity. These were then quality checked using the Agilent TapeStation DNA 1000 tape and DNA HS Qubit. Pooled libraries was taken into sequencing at 2nM and loaded on the NovaSeq 6000 at 400pM with an SP 100 cycle flowcell.

Single cell RNA sequencing analysis

LAM lung scRNAseq data were retrieved from the LAM Cell Atlas with scRNAseq data QC, prefiltering, batch correction and data integration performed as previously described[7]. 65,287 cells from 11 LAM lungs (13 biological replicates) and 43,128 cells from 8 control female lungs[8 9] were included in integrative analysis using Seurat 3[10]. Unbiased cell clustering was performed using the Leiden algorithm[11]. Cell clusters were mapped to cell types based on expression of known cell type marker and signature genes from our previous LAM and normal lung single cell studies[7 12 13]. Automated cell type annotation was done using our previous LAM single cell study and our recently released LungMAP CellRef as references[7 14]. Integrated single cell analysis identified an AT2 cluster consisting of 12,291 LAM AT2 cell and 17,378 control AT2 cells. The Wilcoxon ranksum test was used to compare the gene expression levels between AT2 cells in LAM and control. The fold change (FC) was calculated as the ratio of the average expression level in the AT2 populations in LAM lungs and in normal control lungs. Functional enrichment analysis was performed using ToppGene suite[15]. CellChat[16] analysis was applied on the scRNA-seq data to decipher LAM^{CORE} and LAM AT2 cell communication patterns based on cell type selective expression of ligands secreted from LAM^{CORE} cells and receptors expressed on the surface of LAM AT2 cells. Ingenuity Pathway Analysis (IPA) was used to predict activated upstream regulators using the differentially expressed senescence genes in LAM AT2 cells as input genes.

Bulk RNA-seq analysis

RNA sequencing analyses (RNA-seq) were performed on laser capture microdissection of LAM nodule and the rest of the lung as self-control. RNA-seq data alignment, QC and normalization was done using the RNA-Seq workflow in Partek Flow software (v7. Partek Inc., St. Louis, MO, USA). Human genome hg38 was used as reference. Gene counts were normalized using the median of ratios (i.e., ratio of read count to its geometric mean across all samples before being subjected to a differential expression analysis[17]. Differentially expressed genes (DEGs) were identified using DESeq2 in Partek with the combination criteria of a p-value < 0.05 and absolute fold change >1.5. Genes with mean average count across all

samples < 10 were removed for further analysis. DESeq2 was used to find differentially expressed genes between the laser capture samples and their corresponding rest of lung samples for each clinical measurement group.

Immunohistochemistry

Tissue for immunohistochemistry was obtained from 20 biopsies and four explanted lungs from transplantation and three control female lungs matched for age. Paraffin-embedded lung tissues were dewaxed in Xylene (Merck 214736, UK) and rehydrated with ethanol (100%, 70% each twice, and water). Antigen retrieval was carried out by heating sections sodium citrate buffer or TrisEDTA buffer follow by quenching endogenous peroxidase activity by 3% H2O2. Primary antibodies used were: anti-melanoma associated antigen PNL2 (1:100, Zytomed MSK082-05), mouse monoclonal anti-smooth muscle actin (1:5000 Sigma Clone 1A4, A2547), mouse anti-Ki67
8D5 (1:250, Cell Signaling Technology, No. 9449), P16^{INK4A} Rabbit Polyclonal antibody (1:500, Proteintech No. 10883-1-AP), p21$^{Waf1/Cip1}$ (12D1) Rabbit mAb (1:50 Cell Signaling Technology No: 2947), anti-pro-surfactant protein C antibody Rabbit monoclonal (1:1,000, Abcam, ab90716), CD68 (1/500, Abcam ab213363), TCF21 (1/100 ARG40420) MFAP5 (1/1000, Abcam ab203828) and surfactant protein c/SP-C (H-8) Alexa Fluor 488 conjugated (1/100, Santa Cruz Biotechnology, sc-518029 AF488) Secondary antibodies used were horseradish peroxidase.
(HRP)-conjugated goat anti-rabbit or anti-mouse (ImmPRESS, Vector Laboratories, MP-7452). Detection of primary antibodies was performed using ImmPACT 3,3'-diaminobenzidine (DAB) peroxidase substrate (Vector Laboratories, SK-4105). Sections were counterstained with Mayer's hematoxylin. Fluorescence secondary antibodies used were Goat anti-Rabbit IgG, Alexa Fluor 488 (Invitrogen, A-11008), Goat anti-Mouse IgG, Alexa Fluor 488 (Invitrogen A-11001), Donkey anti-Rabbit Antibody, Alexa Fluor 594 (Invitrogen, A-21207) and Donkey anti-Mouse IgG, Alexa Fluor 594 (Invitrogen, A-21203). Slides were scanned using Hamamatsu Nanozoomer and quantified using QuPath software.
(https://qupath.readthedocs.io/en/stable/docs/tutorials/cell_detection.html). For Fluorescence immunostaining slides were scanned using PhenoImager Fusion (Akoya Biosciences) and QuPath

software were used for quantification. (https://www.protocols.io/view/qupathimmunofluorescence-cell-detection-and-co-lo-e6nvwdmnwlmk/v1)

Immunofluorescence

Organoids were collected after centrifugation at 500G for 5 minutes. Samples were fixed with either 4% paraformaldehyde (PFA) or ethanol based on the primary antibody protocol for 30 min at RT, follow by permeabilization using 0.1% Triton X-100, 0.05% Tween-20 in PBS. Samples were blocked in 3% BSA in PBS. Primary antibodies were incubated at 4 °C overnight and secondary antibodies incubated for 1 hour room temperature and counterstained with DAPI. AT2 Organoids were fixed with PFA for 30 minutes and then released from matrigel by incubating the culture with Corning Cell Recovery Solution (Merck, CLS354253) for 2 hours at 4 °C.

Senescence associated β-galactosidase activity

Senescence associated β-galactosidase was measured in cell cultures using a plate-based fluorescent assay (Cell Signaling, 23833). In tissues β-galactosidase activity at pH 6 was determined using either Senescence β-Galactosidase Staining Kit (cell signaling, 9860) or the CellEvent Senescence Green Detection Kit (Invitrogen, C10850) according to the conditions.

Animal models

TTJ cells (10^6 cells PBS) were injected into the tail vein of female Albino C57BL/6 (B6N-TyrcBrd/BrdCrCrl) with sham injections (PBS) used for control animals[3]. All the mice in this study were female, as LAM affects almost exclusively women. After the TTJ cell–injected untreated group had lost 10% of body weight, the study was terminated in all groups and senescence associated β-galactosidase measured in lung tissue. The project was conducted under Home Office project Personal Project Licence P435A9CF8. A transgenic model mice model, using a conditional *Tsc2* knock-out gene in lung mesenchyme, on a Tbx4LME_Cre background[18], as previously described[9]. Animals were treated with rapamycin (4mg/Kg, i.p.) from birth and lung tissue harvested at 20 weeks. Mice maintained in IVCs

(Tecniplast UK) within a barriered unit illuminated by fluorescent lights set to give a 12 hour light-dark cycle (on 07.00, off 19.00), as recommended in the United Kingdom Home Office Animals (Scientific Procedures) Act 1986. The room will be air-conditioned by a system designed to maintain an air temperature range of 21 ± 2ºC and a humidity of 55% + 10%.

Mice housed in social groups during the procedure with irradiated bedding and provided with autoclaved nesting materials and environmental enrichment.

Western blotting

Nuclear and cytoplasmic protein fractions were separated using a subcellular fractionation protocol as described[19]. Blots were probed with primary antibodies overnight and secondary antibodies for one hour room temperature. Primary antibody P16-INK4A Rabbit Polyclonal antibody (1:100, Proteintech No. 10883-1-AP), p21 Waf1/Cip1 (12D1) Rabbit mAb (1:100 Cell Signaling Technology No: 2947) and β-actin Rabbit mAb (Cell signaling, 4970) was used as a loading control. Clarity Max Western ECL Substrate was used for HRP conjugated (BIO-RAD 1705062) for detecting the protein bands. In all cases, whole lung lysates were used for protein analysis.

IL6 ELISA

Conditioned media were passed through a 0.2μM filter and IL-6 quantified using a Human IL-6 DuoSet ELISA (R and D, DY206) according to the manufacturer's protocol. The optical density measured at 450 nm with wavelength correction of 540 nm. Serum IL6 was determined using the same kit in 88 LAM patients and 13 control subjects.

Supplementary Results

Supplementary table 1. Clinical features of patient samples for laser capture microdissection.

Patient characteristics				Disease at time of tissue sample			Lung function loss		Disease features				
Age(yrs)	Race	Sporadic or TSC -LAM	Menopause	FEV_1	TL_{CO}	Diseaseduration (months)	ΔFEV_1	ΔTL_{CO}	Presentation	Everhadpneumothorax	Angiomyolipomapresent	Lymphaticinvolvement	VEGFD(pmol/ml)
49	white	sporadic	yes	18.7	33.4	228	-0.105	-0.370	angiomyolipoma	yes	yes	no	666
49	white	sporadic	no	77.5	60.8	240	NA	NA	pneumothorax	yes	no	no	2323
20	African	TSC-LAM	no	50.4	56.0	3	-0.075	-0.163	pneumothorax	yes	yes	no	289
30	white	TSC-LAM	no	76.0	57.1	1	-0.092	-0.132	dyspnoea	yes	yes	no	1028
69	white	sporadic	yes	83.1	45.3	228	NA	NA	pneumothorax	yes	no	no	397
44	white	sporadic	yes	49.5	40.0	72	-0.029	-0.082	pneumothorax	yes	yes	no	1326
31	white	sporadic	no	94.9	67.3	12	-0.142	-0.247	angiomyolipoma	yes	yes	no	702
28	white	sporadic	no	78.4	56.8	1	NA	NA	other respiratory	no	no	yes	4287
49	white	TSC-LAM	no	69.8	68.8	60	-0.166	-0.275	chance finding	yes	yes	no	NA
37	Iranian	sporadic	no	66.3	72.5	1	-0.063	-0.261	pneumothorax	yes	yes	yes	1163
48	white	sporadic	yes	78.0	73.0	12	NA	NA	pneumothorax	yes	no	no	3765
21	white	TSC-LAM	no	35.2	37.9	12	-0.089	-0.656	angiomyolipoma	yes	yes	no	3590
40	white	sporadic	no	89.6	74.9	35	-0.258	-0.731	pneumothorax	yes	no	no	586
36	white	sporadic	no	76.0	54.7	72	-0.014	-0.133	pneumothorax	yes	no	no	645
30	West Indian	sporadic	no	67.9	37.3	48	NA	NA	dyspnoea	no	no	no	967
41	white	sporadic	no	87.2	81.8	6	-0.028	-0.094	pneumothorax	yes	no	no	416
30	West Indian	sporadic	no	67.9	37.3	48	NA	NA	dyspnoea	no	no	no	967
21	white	TSC-LAM	no	35.2	37.9	12	-0.089	-0.656	angiomyolipoma	yes	yes	no	3590
55	white	sporadic	yes	91.5	90.3	23	-0.059	-0.155	dyspnoea	yes	no	no	834

FEV_1 / TL_{CO} = percent predicted value for forced expiratory volume in 1 second / transfer of lung carbon monoxide at the time of tissue biopsy. ΔFEV_1 = rate of loss of FEV_1 (litres/year). ΔTL_{CO} = rate of loss of TL_{CO} (mmol/min/kPa/year). VEGF-D = serum vascular endothelial growth factor type D. NA = not available

Supplementary table 2. 100 most upregulated genes assessed by bulk RNA sequencing in laser capture isolated LAM nodules from 19 patients with LAM compared with normal lung tissue. The full dataset is available at GEO (accession number: GSE265851). Differentially expressed genes (DEG) between LAM vs control were identified using DESeq241, which performs a two-sided test. The y-axis shows p-value (<0.05) and the x-axis shows Log2(FC) (FC>1.5). FDR was calculated but not used for the DEG selection due to the high sample variations.

Gene symbol	P-value (LAM vs. Control)	Fold change (LAM vs. Control)	LSMean (LAM) (LAM vs. Control)	LSMean (Control) (LAM vs. Control)
MMP11	0.009964729	269.2890793	269.2890793	1
ZNF324	0.002618641	156.1631409	156.1631409	1
ESRP2	0.013179556	125.7658885	182.5727108	1.451687043
MRGPRF	0.002686885	115.4989799	115.4989799	1
B3GNT8	0.046429956	97.10326966	97.10326966	1
TRIM63	0.026479265	94.01686195	151.8733889	1.615384579
FN3KRP	0.009515228	88.32529251	88.32529251	1
SLC18B1	0.01446082	85.56842825	85.56842825	1
ZBTB47	4.58475E-06	85.18364266	203.1302279	2.384615421
ARIH2OS	0.04832264	85.09948858	85.09948858	1
PDE6B	0.021687956	82.67230709	82.67230709	1
KLHL6	0.03239884	79.88639557	79.88639557	1
TTC39A	0.034483036	79.08945967	79.08945967	1
KIF21B	0.014195162	77.25483045	77.25483045	1
ABCB5	0.047272775	77.06538613	77.06538613	1
MINPP1	0.018562675	76.83336032	76.83336032	1
TRPV4	0.029434481	75.75740846	75.75740846	1
TMEM273	0.046352612	74.445846	74.445846	1
AGRP	0.009042088	74.1212295	74.1212295	1
WASH8P	0.005883626	73.82564502	111.153656	1.505623905
TFEB	0.044180349	70.01291569	70.01291569	1
HBP1	0.044560805	69.68997832	104.5385331	1.500051164
LY96	0.029953598	67.21379853	67.21379853	1
ZNF124	0.004484127	66.91309454	66.91309454	1
ITGBL1	0.016009334	66.07936179	66.07936179	1
CACNA1C	0.006254952	65.04068262	265.1658599	4.076923077
COL15A1	0.037650534	64.33278108	103.9221825	1.615384579
ABCC3	0.000286535	61.73773608	147.2207575	2.384615421
TMX3	0.008167493	58.75177363	92.35557856	1.571962391
TMEM254	0.012666949	54.67523868	88.32153741	1.615384579
HERPUD2	0.004503543	54.11452936	364.2324251	6.730769524
ZNF468	0.021826726	53.41839874	86.29125754	1.615384579

PUSL1	0.010066043	52.65842065	111.9494087	2.125954545
WDR46	0.029532138	52.09170221	52.09170221	1
BTBD3	0.043465562	49.86581414	49.86581414	1
MMP7	0.02045438	48.35176944	48.35176944	1
PSD4	0.002114871	48.28541983	232.3417809	4.811841373
PTPN22	0.045416904	48.1705179	77.81391177	1.615384579
USP31	0.032651053	47.93020929	77.42572095	1.615384579
FAM13B	0.028659848	47.43003036	47.43003036	1
SAMD4A	0.043305301	45.7661526	45.7661526	1
EEPD1	0.045044065	44.43364912	44.43364912	1
NIPSNAP3A	0.002418215	43.38702904	68.2027779	1.571962391
REEP1	0.036820016	42.91731216	42.91731216	1
MYBBP1A	0.037644631	42.18050104	61.64842344	1.461538434
RAB4A	0.014934588	41.66483349	67.3047295	1.615384579
TUBA3FP	0.043446582	41.40106778	41.40106778	1
POP4	0.033767083	41.28777134	41.28777134	1
SLAMF1	0.02971143	41.23838938	66.61585825	1.615384579
C2orf74	0.029066569	41.09145329	132.7570028	3.230769228
DNAJC13	0.042834999	40.01053265	40.01053265	1
NUDCD2	0.004887862	39.87728419	83.69774275	2.098882721
ZNF526	0.023840127	38.75067583	60.91460504	1.571962391
NDFIP2	0.024414455	38.35969617	82.62088266	2.153846117
FNIP1	0.00152943	37.38129118	80.36950795	2.149992828
MIR4458HG	0.02121217	36.43005711	36.43005711	1
PAIP1	0.009413994	36.0556155	97.07284177	2.692308547
MTFP1	0.044782069	35.6642344	57.61145426	1.615384579
CASK	0.018776248	35.35325218	35.35325218	1
UBQLN2	0.047269592	34.63151664	34.63151664	1
RRN3	0.001842464	34.27916462	73.83204562	2.153846117
ZNF780B	0.038688513	33.96796958	33.96796958	1
RASGRF1	0.024198664	33.23967821	71.59315185	2.153846117
ANKS1B	0.030157088	32.87523363	32.87523363	1
AZIN1	2.86268E-05	32.69527222	96.82830605	2.961538458
PRPS2	0.014718588	32.18198898	51.98628872	1.615384579
MPI	0.00526314	31.84420849	144.2727916	4.530581804
CPED1	0.004182107	31.61256011	51.0664421	1.615384579
BAG3	0.018451605	31.3730299	31.3730299	1
MOCOS	0.035783658	30.14709246	48.69914826	1.615384579

GMCL1	0.000880407	30.01472068	202.0221673	6.730769524
COA4	0.001196928	29.97050652	80.68985087	2.692308547
PRKCA	0.009781408	28.9454801	77.93016348	2.692308547
HERC2	0.000483275	28.77297612	85.21227532	2.961538458
LRP2	0.047726953	27.60730878	27.60730878	1
CBWD3	0.034784119	27.56930914	42.23065401	1.531799502
CREG1	0.0013251	27.46807189	132.1720048	4.811841373
ZNF330	0.016750249	27.20878204	58.60352955	2.153846117
SCAI	0.014609859	27.04236583	190.3371349	7.038479402
COX18	0.043693342	26.42418025	78.25622603	2.961538458
BRCC3	0.03302363	25.79044039	25.79044039	1
CENPC	0.049512851	25.51200085	60.83631067	2.384615421
KCNN4	0.020488461	25.43452669	54.78205654	2.153846117
LOC101930085	0.04864007	24.65906918	39.83388009	1.615384579
MRPS22	0.020083362	24.58514622	24.58514622	1
MEPCE	0.01570748	24.05256965	215.1090363	8.943287116
TMEM131	0.043581672	23.91394658	51.50696098	2.153846117
MFSD1	0.020894764	23.61842752	76.30568886	3.230769228
DERL1	0.001134589	23.39209571	101.6656416	4.346153626
NOM1	0.033069138	22.78797241	54.34055043	2.384615421
SRRD	0.043624123	22.48154231	33.72976381	1.500331398
TXNRD2	0.041359093	22.43136662	74.22392505	3.308934597
AP1S3	0.000821575	22.26630472	705.0110887	31.66268932
NSUN3	0.023614577	21.79710146	83.83500482	3.846153809
NTM	0.013592975	21.34528967	701.0215481	32.84197868
SH3PXD2B	0.027826486	21.22578253	142.8658501	6.730769524
VIRMA	0.005658337	20.89635237	61.88535117	2.961538458
URB1	0.030636798	20.8855265	49.14393648	2.353014011
NUFIP1	0.04922981	20.10746966	32.4812964	1.615384579
BRD8	0.003233504	19.70280121	68.11473792	3.457109332

Supplementary table 3. AT2 cell senescence-related genes from the SenMayo panel upregulated in LAM. DEG were identified using Wilcoxon Rank-Sum Test (two- sided test) with p value <0.05 and fold change >1.5. No multiple comparison correction was applied for DEG identification. FDR <10% was applied for pathway and gene set enrichment analysis.

**Gene**	**P value**	**Fold change** (average log2)	**LAM**	**Control**
CCL20	8.26E-221	1.3586	0.227	0.099
NAMPT	0	1.268706	0.718	0.539
CXCL2	0	1.246963	0.797	0.591
CCL2	0	1.153067	0.201	0.041
JUN	0	1.143426	0.842	0.772
C3	0	1.139472	0.84	0.456
ICAM1	0	1.135248	0.651	0.483
CXCL1	0	1.077755	0.293	0.097
EGR1	0	1.072418	0.694	0.525
SOD2	0	1.055837	0.769	0.603
ERBB4	1.68E-110	1.041615	0.182	0.099
JUNB	0	1.02069	0.829	0.687
RRAD	0	0.946155	0.328	0.118
DUSP1	0	0.927585	0.894	0.658
ID1	0	0.912205	0.7	0.511
SOX5	4.21E-265	0.909006	0.122	0.023
MAPK10	0	0.870746	0.224	0.058
KLF4	0	0.82082	0.326	0.084
CD36	0	0.820471	0.504	0.246
CXCL8	7.90E-165	0.775338	0.252	0.133
CXCL3	1.91E-242	0.697865	0.284	0.133
ID3	0	0.687593	0.393	0.198
HSPA1A	2.65E-168	0.639386	0.439	0.291
ID2	7.55E-244	0.63303	0.465	0.305
XBP1	0	0.626724	0.83	0.722
TNFRSF1A	0	0.584415	0.57	0.304
UBC	0	0.581075	0.906	0.877
RAC1	0	0.577818	0.806	0.608
HSPB1	0	0.558992	0.801	0.657
PDZK1IP1	0	0.538204	0.481	0.235
CDKN1A	3.81E-273	0.531578	0.392	0.214
HIF1A	1.89E-295	0.494481	0.535	0.338
PRKD1	4.29E-130	0.476476	0.125	0.049
CEBPB	0	0.450393	0.679	0.462
TSC22D1	7.17E-226	0.445661	0.839	0.719
BRAF	2.85E-73	0.430835	0.272	0.189
LMNA	2.58E-113	0.426885	0.698	0.62
IL32	0	0.418719	0.227	0.062
SRSF2	1.94E-307	0.409407	0.65	0.469

MIF	2.57E-49	0.406996	0.63	0.627
FGFR2	2.70E-25	0.393774	0.244	0.198
TOP1	1.03E-148	0.390084	0.796	0.704
CD55	3.73E-48	0.371751	0.705	0.651
PPP1R13B	9.69E-103	0.367679	0.21	0.121
SMC5	2.97E-120	0.356548	0.363	0.241
ERN1	1.84E-150	0.343214	0.379	0.237
NSMCE2	5.96E-45	0.336313	0.183	0.128
FBP1	8.87E-233	0.336221	0.566	0.386
KDM2A	2.80E-92	0.330557	0.238	0.149
SCMH1	1.61E-103	0.324639	0.137	0.065
WWTR1	5.67E-23	0.318509	0.439	0.38
DUOXA1	6.26E-17	0.314355	0.502	0.463
SPRY4	6.71E-193	0.312054	0.37	0.214
TEAD1	3.75E-97	0.311361	0.314	0.208
DYRK1A	6.33E-39	0.293529	0.225	0.168
ZFP36L1	1.83E-32	0.292388	0.764	0.762
ATXN1	0.0065199	0.286198	0.185	0.179
IGFBP4	1.60E-182	0.272649	0.406	0.25
BABAM2	1.12E-40	0.263499	0.224	0.166
SMURF2	4.42E-55	0.260193	0.234	0.163
PTTG1	1.97E-228	0.258005	0.211	0.082
SOCS1	3.43E-297	0.257965	0.211	0.066
CAT	1.00E-169	0.257032	0.689	0.528
SRSF3	7.21E-132	0.255689	0.74	0.628
THRB	2.24E-56	0.255262	0.146	0.089
GPATCH8	7.30E-35	0.252401	0.195	0.144
PHC2	2.52E-106	0.250933	0.312	0.205

LAM p21

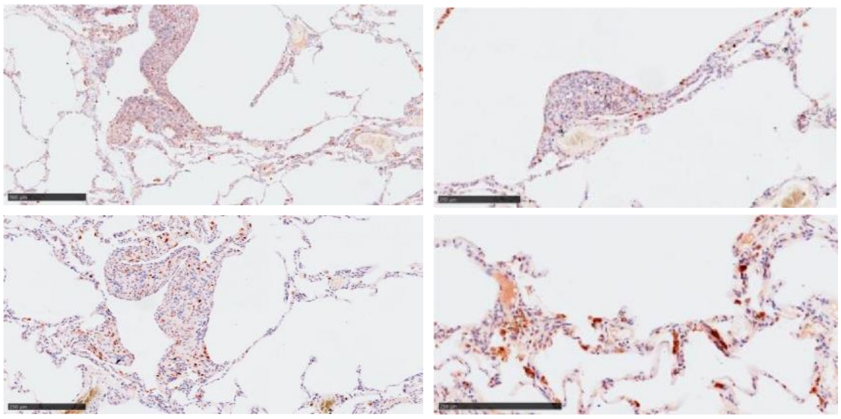

LAM p16

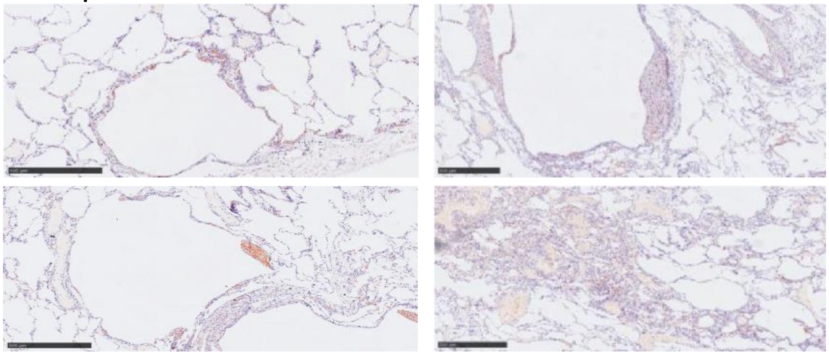

Normal lung p21 isotype control

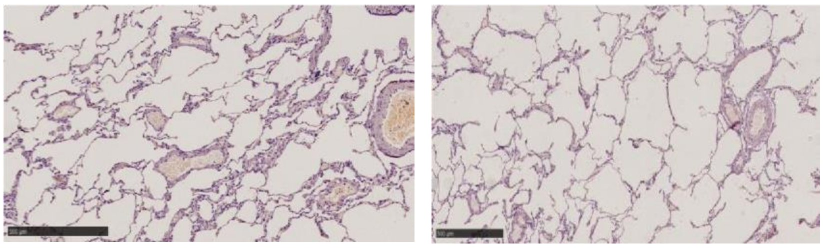

Normal lung p16 isotype control

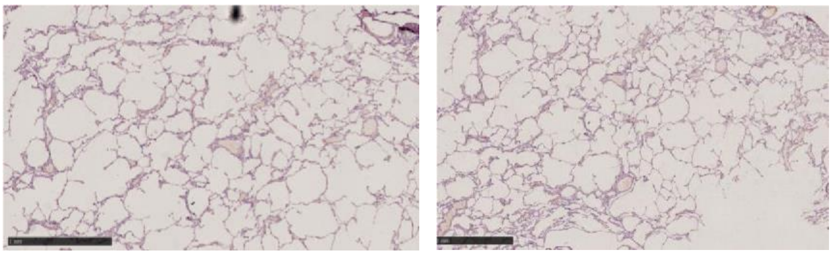

Supplementary Figure 1. Immunohistochemical staining of LAM and control healthy lung tissue. Representative images of senescence (p16 and p21), markers (brown) in LAM and healthy control lung tissue (n=21). Isotype control panels have undergone the same protocol with the primary antibody substituted with a non-specific antibody of the same isotype (n=21)

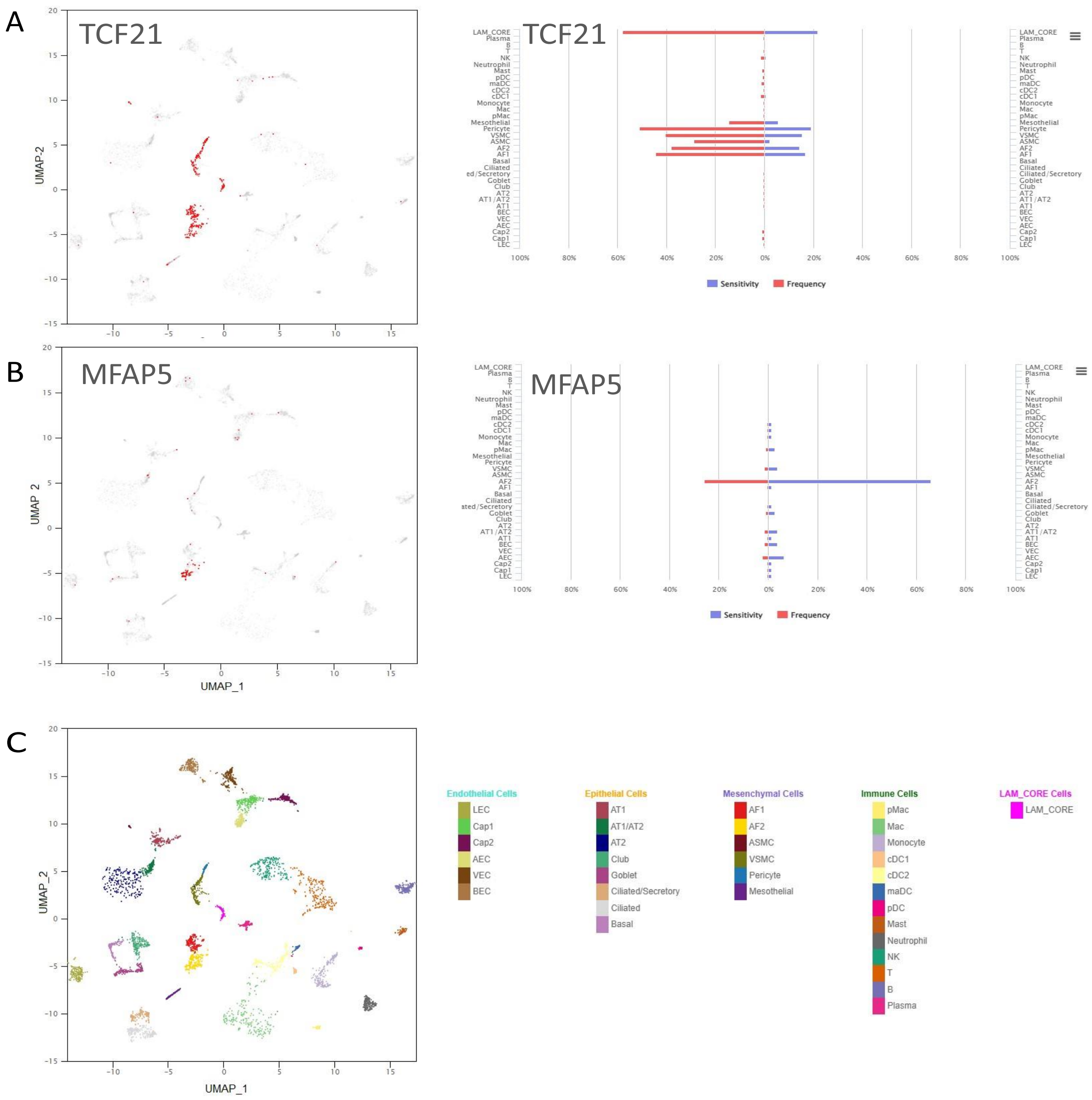

Supplementary Figure 2. Fibroblast markers genes. Single cell sequencing of LAM lung tissues Showing expression of TCF21 and *MFAP5* in LAM lung populations using the LAM cell atlas[2] . (A) Left panel shows expression of positive cells for *TCF21* in each cluster, right panel shows the fold change and significance for LAM compared with control lung. (B) Left panel shows *MFAP5* expression in each cluster, right panel shows the fold change and significance for LAM compared with control lung. (C) panel shows cell types present in LAM lung samples. AF=alveolar fibroblast 1, AF2=alveolar fibroblast 2, VSMC=Vascular smooth muscle cells.

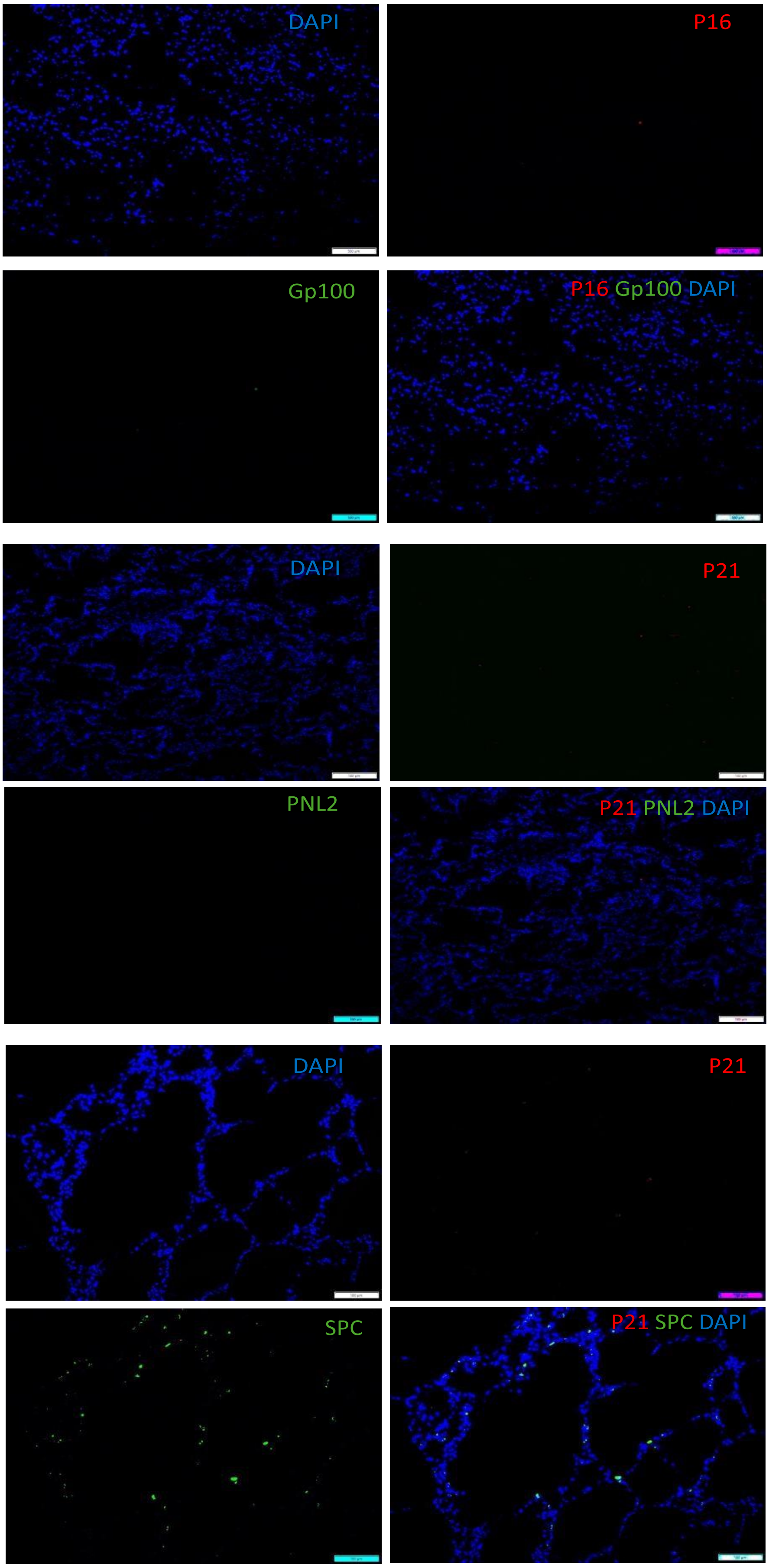

Supplementary Figure 3. Immunohistochemical Staining of control healthy lung tissue. Representative images of senescence (p16 and p21), Alveolar type 2 cell (SPC and LAM cell (GP100 and PNL2) markers (n=5)

A

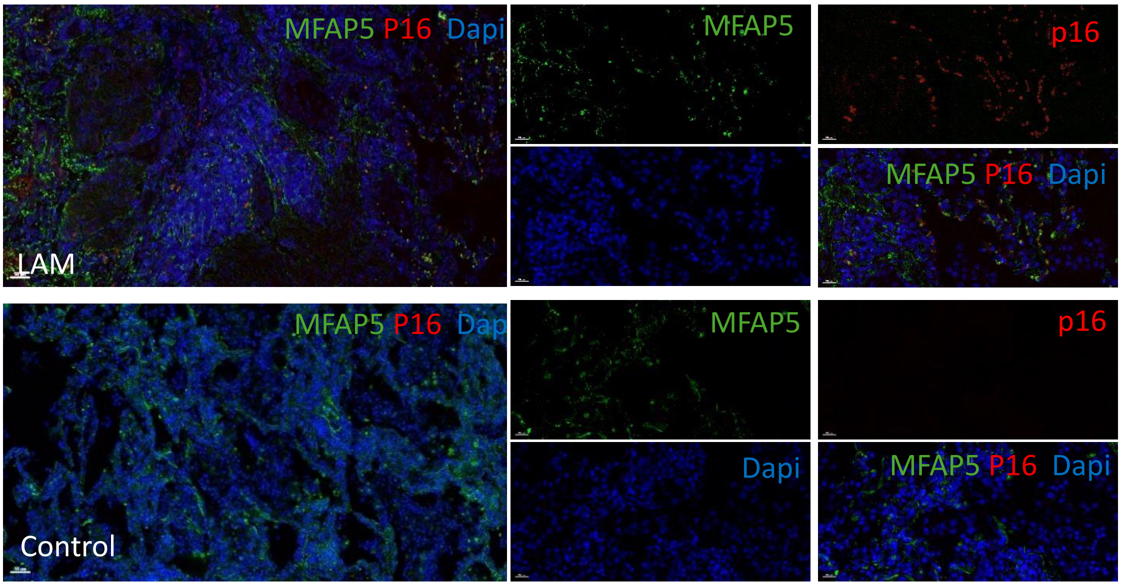

B

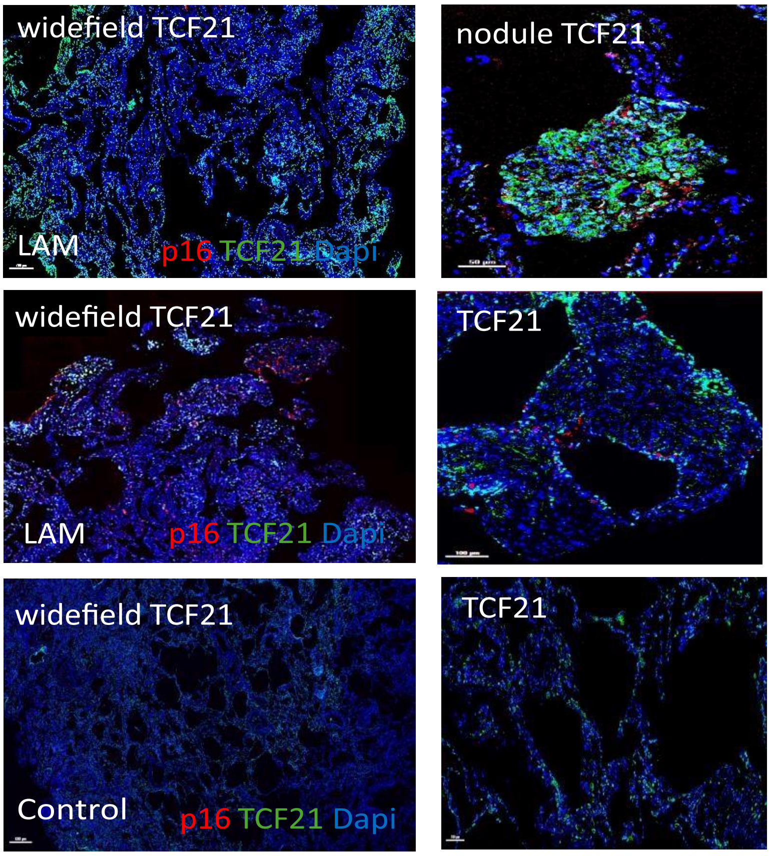

Supplementary figure 4. LAM associated fibroblast markers and senescence.
(A) Representative image of biopsy samples from a patient and control
showing colocalization of MFAP5 and p16. and control lungs. LAM (n=8 each, ≥3 replicate) and control lungs (n=3, ≥3 replicates) (B) Representative widefield and close-up images of dual label
immunohistochemical staining of mesenchymal cell marker TCF21 in
LAM and control LAM (n=8 each, ≥3 replicate) and control lungs (n=3, ≥3 replicates) .

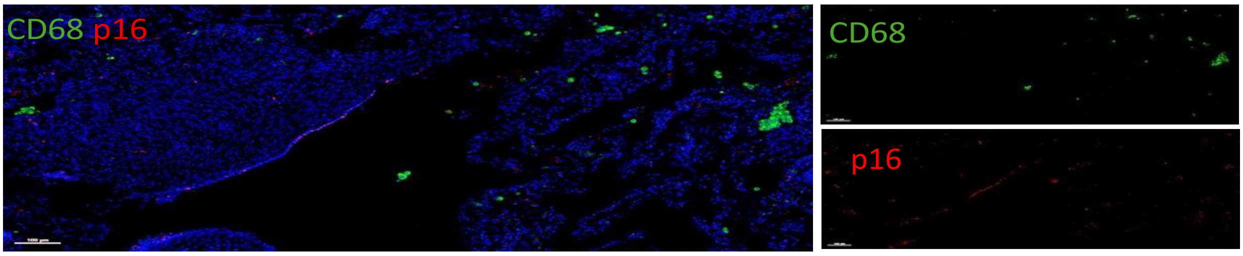

Supplementary figure 5. Representative image of biopsy samples from individuals with LAM co-immunostained for the macrophage marker CD68, p16 and Dapi (n=11).

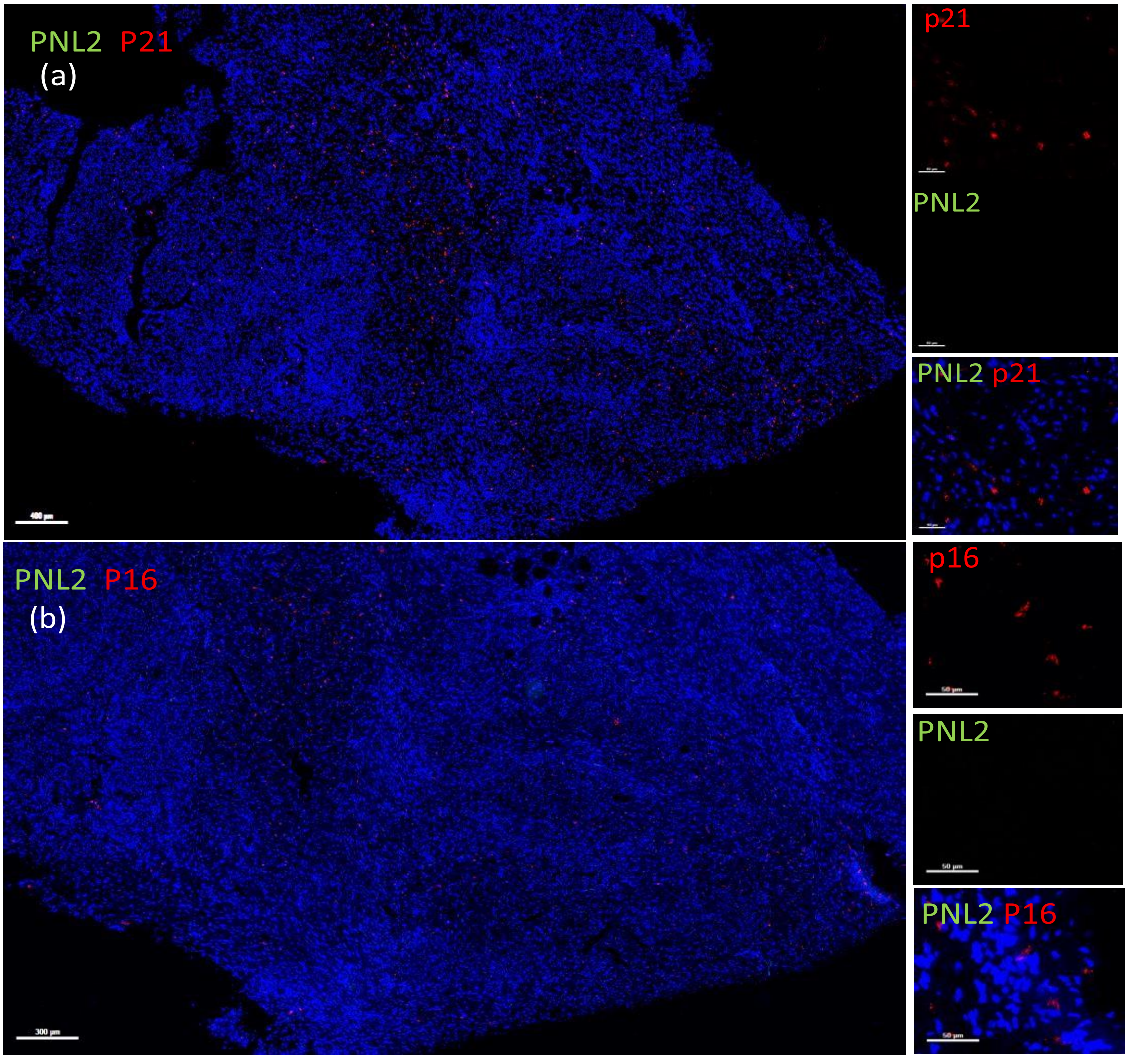

Supplementary figure 6. Expression of p21 and p16 in angiomyolipoma. Four separate angiomyolipomas from patients with sporadic LAM were immunostained for (a) p21 PNL2 (b) p16 and PNL2. (c) p16 and p21 positive cells were quantitated for each tumour as the percentage of the total cell count in the region of interest (n=4). Two tailed unpaired T Test was used to analyse the data P= 0.181. Source data are provided as a Source Data file.

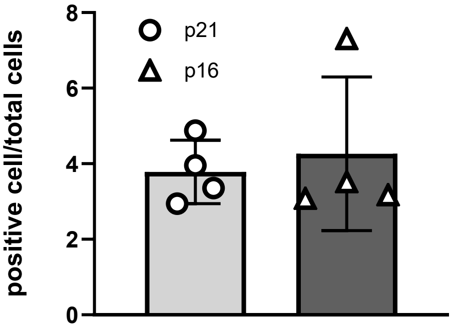

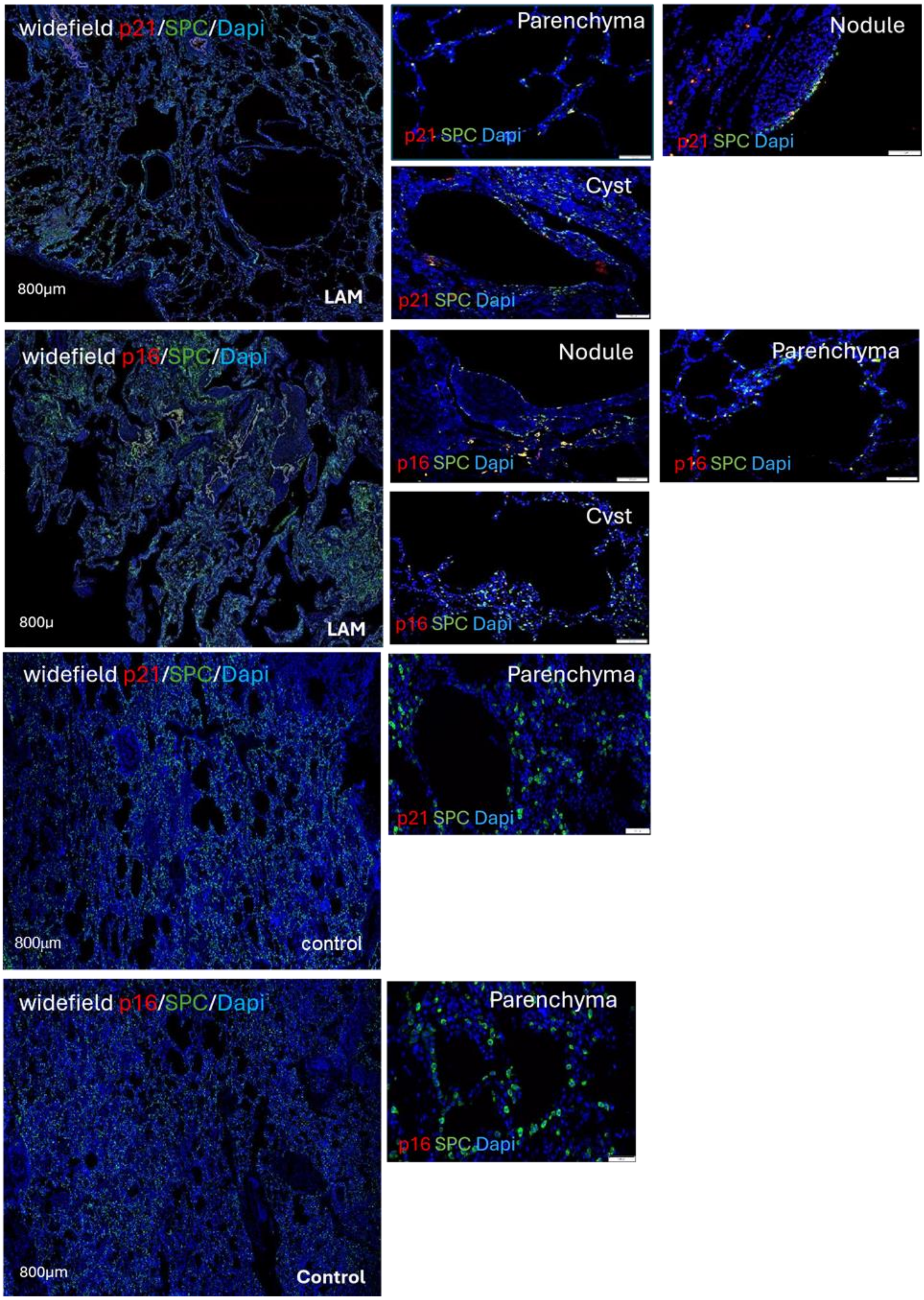

Supplementary figure 7. AT2 cells are senescent in LAM, but not in control. Representative widefield and close up images of dual label immunohistochemical staining of p21/p16 and the AT2 cell marker surfactant protein C (SPC) around LAM nodules, in the walls of lung cysts and normal areas of lung parenchyma and in parenchyma of control subjects. LAM (n≥9, 5 independent images per lung) and control (n=5 and 5 independent images per lung) lungs.

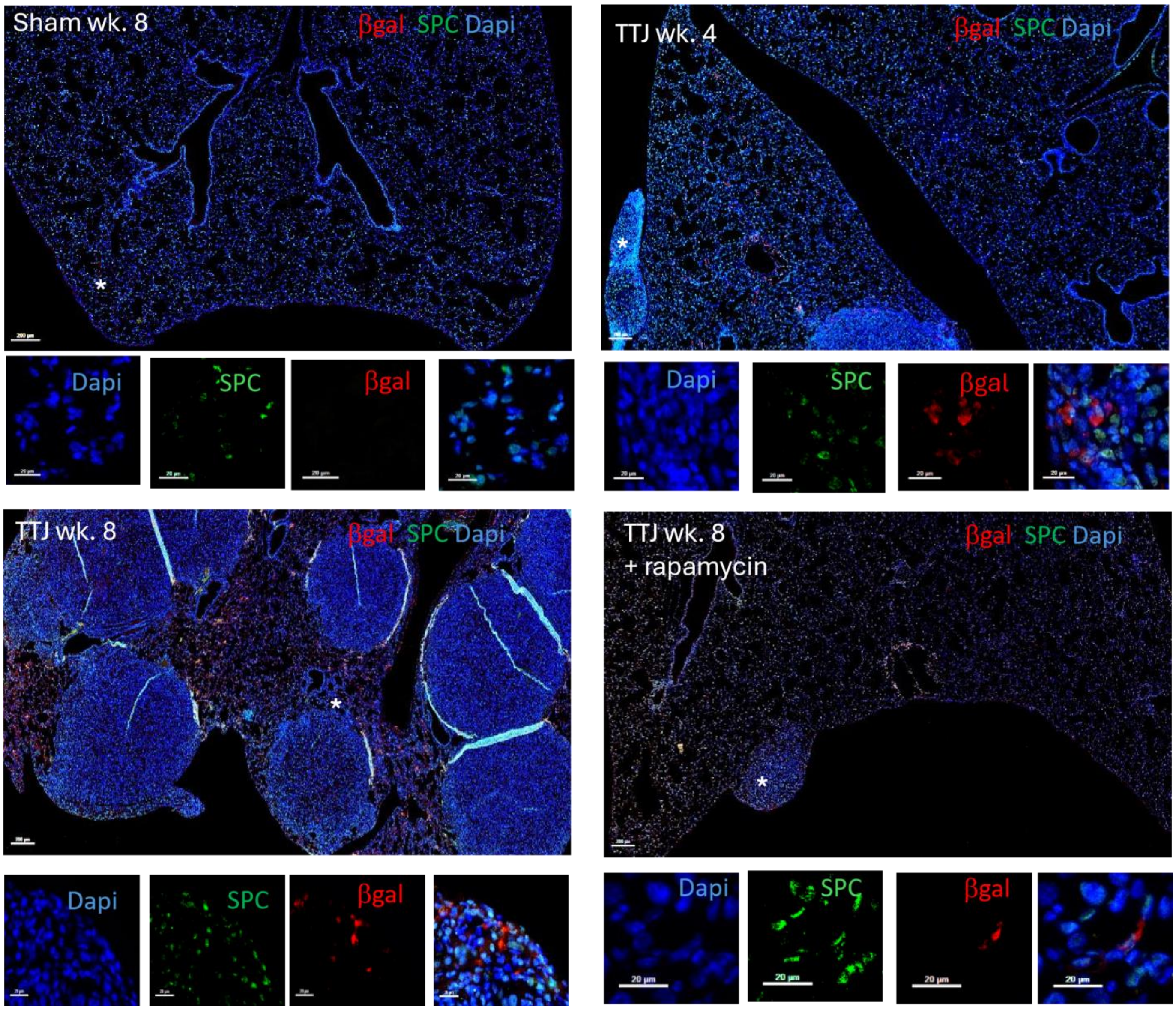

Supplementary figure 8. Expression of βgal and SPC in murine model. TTJ cells or saline (sham) were injected into the tail veins of albino C57BL/6 mice, animals were treated with rapamycin or vehicle two weeks after cell injection. Immunostaining with SPC and beta -galactosidase (βgal) (n=3 / group, 6 independent images per lung).

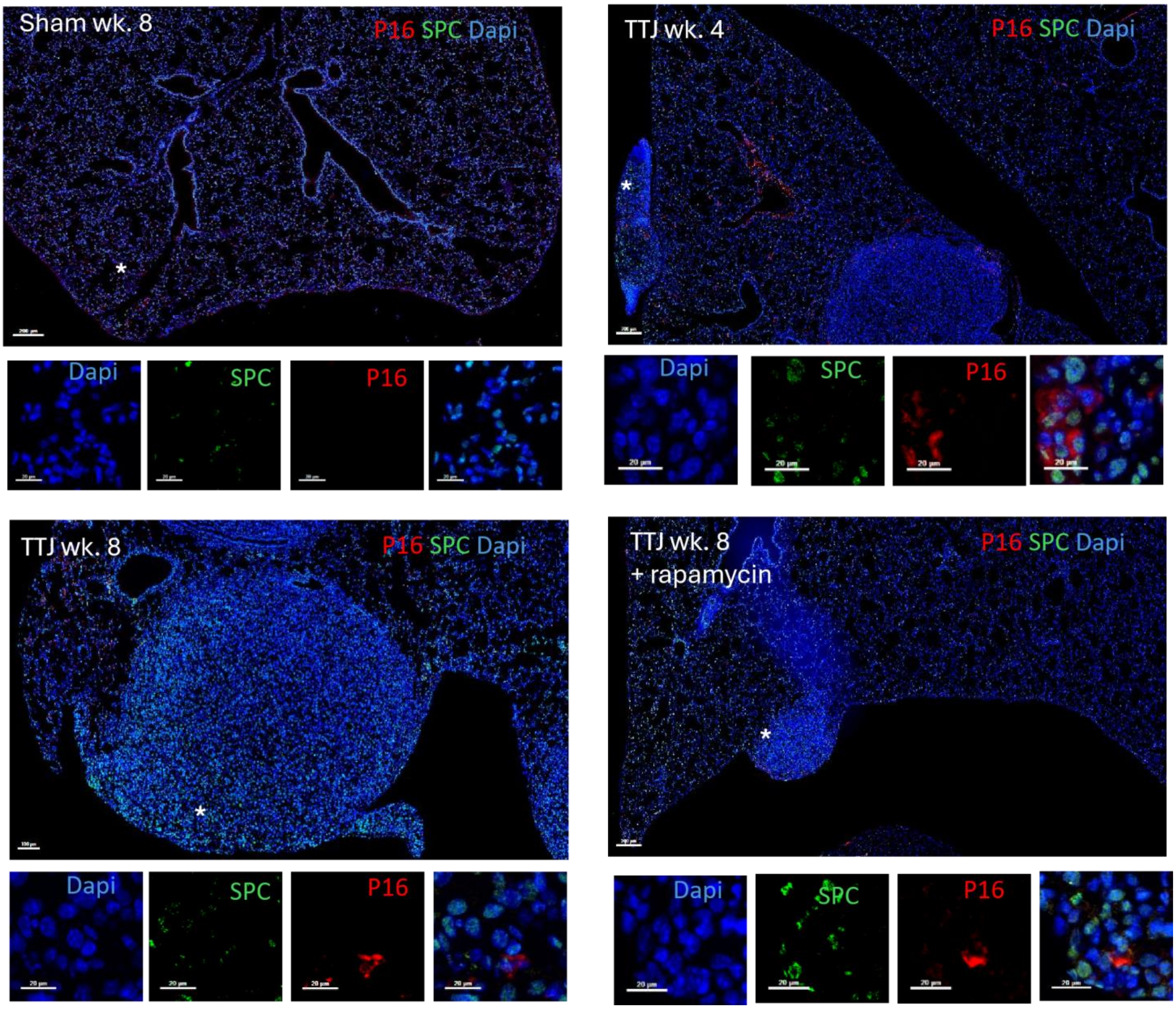

Supplementary figure 9. Expression of p16 and SPC in murine model. TTJ cells or saline (sham) were injected into the tail veins of albino C57BL/6 mice, animals were treated with rapamycin or vehicle two weeks after cell injection. Immunostaining with SPC and p16 (n=3 / group, 5 independent images per lung).

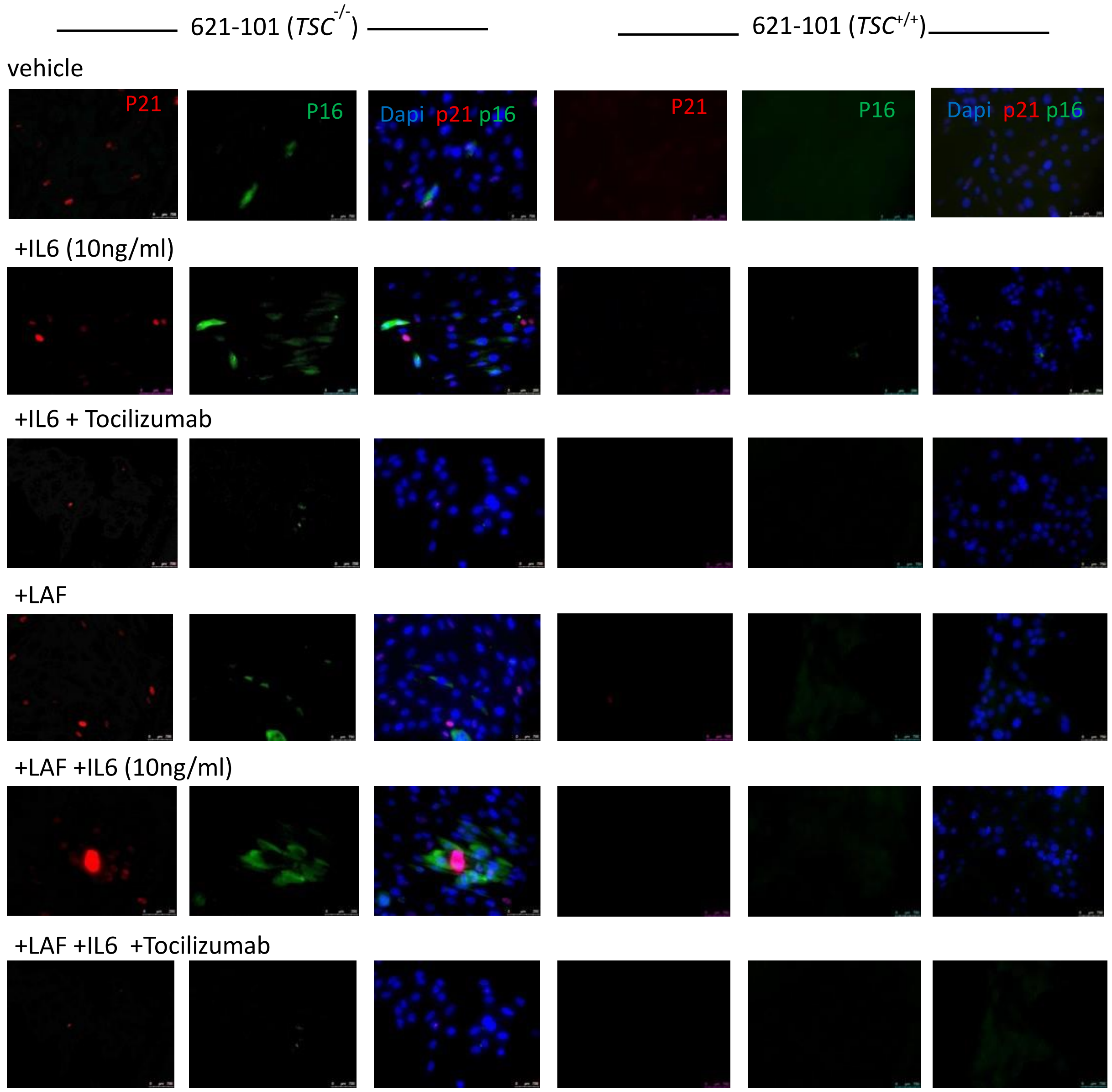

Supplementary figure 10. Immunocytochemistry of 621-101 and 621-103 cells for p16 and p21 cultured in low serum conditions for over 14 days. IL6 induces p16 and p21 in an mTOR dependent manner which is amplified by the presence of LAM associated fibroblasts (LAF). This experiment was repeated 4 times independently with ≥3 replicates with similar results.

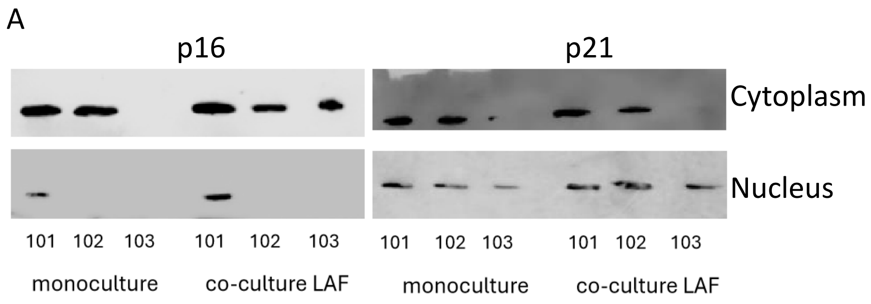

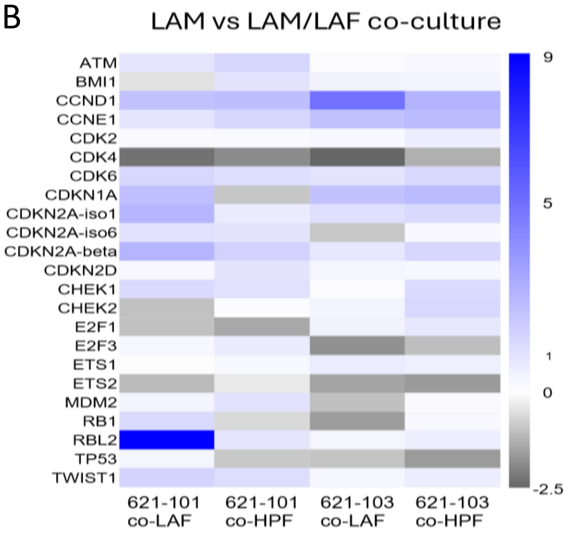

Supplementary Figure 11. Markers of senescence in 621 cells and the effect of *TSC2*. (A) Western blot of nuclear and cytoplasmic fractions of *TSC2*$^{-/-}$ 621-101, *TSC2*$^{-/-}$ 621-102 and *TSC*$^{+/+}$ 621-103 cels alone or co-cultured with LAFs probed for p16 and p21.

(B) Heat map of senescence associated genes analysed by bulk RNA sequencing in *TSC*$^{-/-}$ 621-101 and *TSC* $^{+/+}$ 621-103 cells alone or co-cultured with either LAFs or normal human pulmonary fibroblast (HPF).

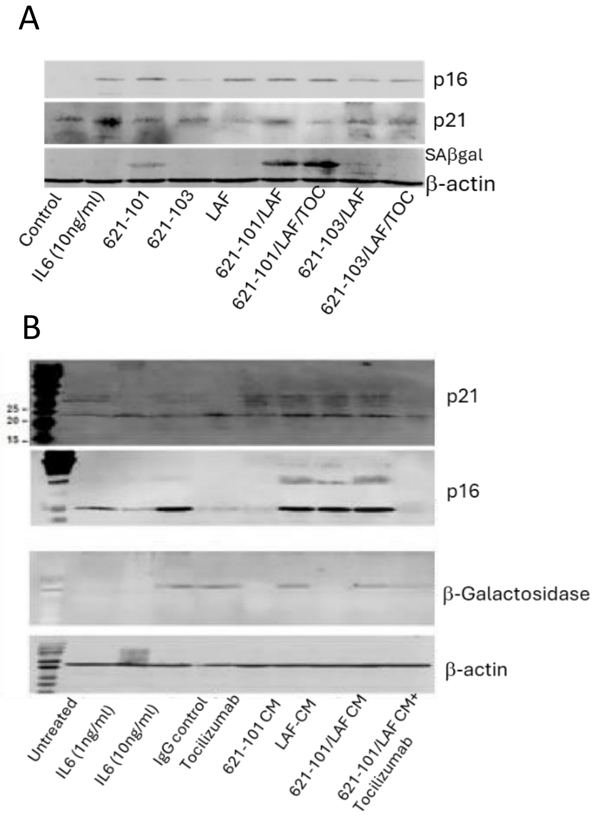

Supplementary Figure 12. IL6 secretion and senescence *in vitro*.

(A) Western blots showing expression of p16, p21, SAβgal protein expression by AT2 cell organoids over 14 days co-cultured with *TSC2*$^{-/-}$ 621-101 cells, *TSC2*$^{+/+}$ 621-103 cells and LAF in mono or co-cultures as described and the effect of Tocilizumab (Toc). Beta actin is used as a loading control.

(B) Western blot showing expression of p16, p21, senescence associated β- gal(SAβgal)protein expression by A549 cells over 14 days co-cultured with *TSC2*$^{-/-}$ 621-101 cells and LAM associated fibroblast (LAF) conditioned media (CM) in mono or co-cultures and the effect of Tocilizumab. β-actin is used as a loading control.

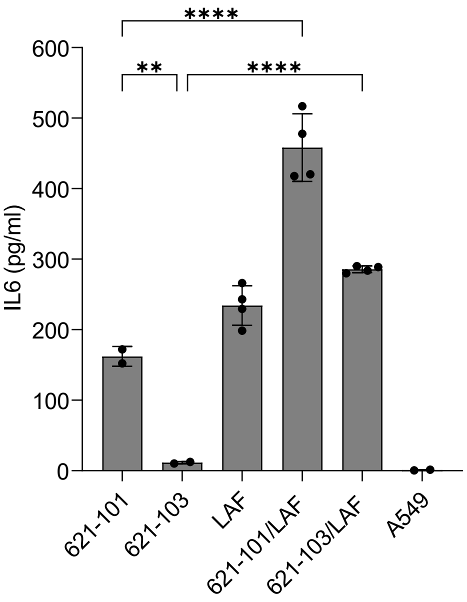

Supplementary Figure 13. IL6 secretion and senescence *in vitro*. (A) Secretion of IL6 by *TSC2*$^{-/-}$ 621-101 cells, *TSC2*$^{+/+}$ 621-103 cells and LAM associated
fibroblasts (LAF), LAM cell/LAF cocultures and epithelial A549 cells analysed by 2-way ANOVA. (n=3, 2 replicates).

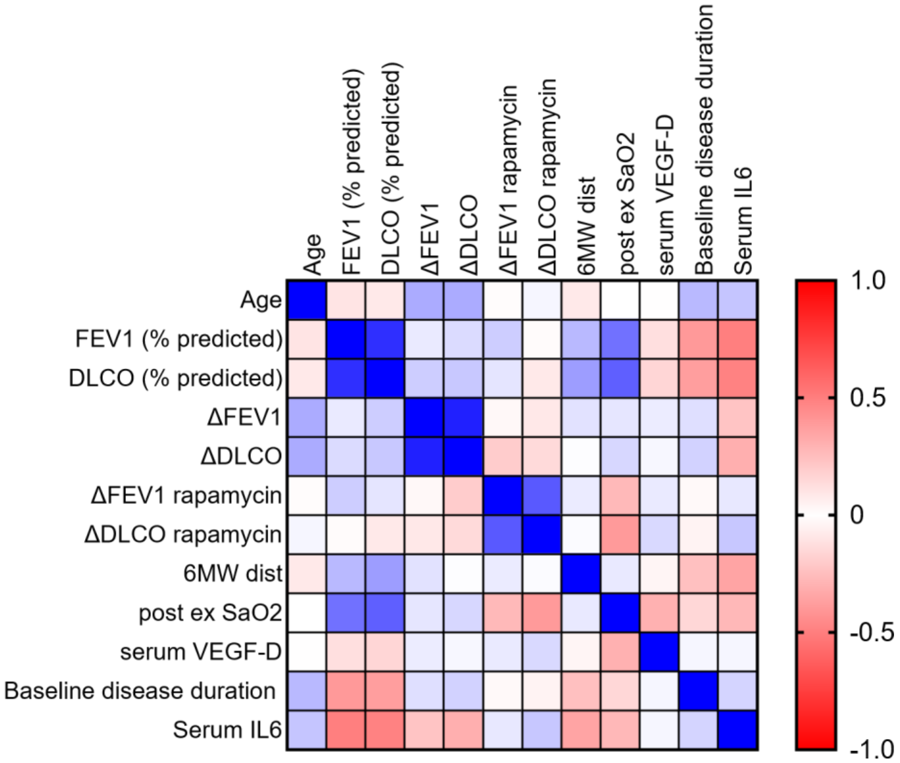

Supplementary Figure 14. Pearson correlation network of clinical parameters and serum IL6 levels in 88 women with LAM. Legend shows correlation coefficient. FEV_1 = forced expiratory volume in 1 second, DL_{CO} = Lung diffusion of carbon monoxide, ΔFEV_1 = rate of loss of FEV_1 during follow up, ΔDL_{CO} = rate of loss of DL_{CO} during follow up, ΔFEV_1 rapamycin = rate of loss of FEV_1 in patients treated with rapamycin during follow up, ΔDL_{CO} rapamycin = rate of loss of DL_{CO} in patients treated with rapamycin during follow up, 6MW dist = six minute walk distance, post ex SaO2 = lowest oxygen saturation during the sixminute walk test, baseline disease duration = time from first symptom attributable to LAM until measurement of serum IL6.

Supplementary References

1. McCormack FX, Gupta N, Finlay GR, et al. Official American Thoracic Society/Japanese Respiratory Society Clinical Practice Guidelines: Lymphangioleiomyomatosis Diagnosis and Management. *American Journal of Respiratory and Critical Care Medicine* 2016;194(6):748-61. doi: 10.1164/rccm.201607-1384ST
2. Yu J, Astrinidis A, Howard S, et al. Estradiol and tamoxifen stimulate LAM-associated angiomyolipoma cell growth and activate both genomic and nongenomic signaling pathways. *Am J Physiol Lung Cell Mol Physiol* 2004;286(4):L694-700.
3. Babaei-Jadidi R, Dongre A, Miller S, et al. Mast Cell Tryptase Release Contributes to Disease Progression in Lymphangioleiomyomatosis. *Am J Respir Crit Care Med* 2021 doi: 10.1164/rccm.202007-2854OC [published Online First: 2021/04/22]
4. Kyungtae L, Eimear NR, Dawei S, et al. A novel human fetal lung-derived alveolar organoid model reveals mechanisms of surfactant protein C maturation relevant to interstitial lung disease. *bioRxiv* 2023:2023.08.30.555522. doi: 10.1101/2023.08.30.555522
5. Lim K, Donovan APA, Tang W, et al. Organoid modeling of human fetal lung alveolar development reveals mechanisms of cell fate patterning and neonatal respiratory disease. *Cell Stem Cell* 2023;30(1):20-37.e9. doi: https://doi.org/10.1016/j.stem.2022.11.013
6. Lim K, Rawlins EL. Protocol for the derivation and alveolar type 2 differentiation of late-stage lung tip progenitors from the developing human lungs. *STAR Protoc* 2024;5(3):103201. doi: 10.1016/j.xpro.2024.103201 [published Online First: 20240718]
7. Guo M, Yu JJ, Perl AK, et al. Single-Cell Transcriptomic Analysis Identifies a Unique Pulmonary Lymphangioleiomyomatosis Cell. *American journal of respiratory and critical care medicine* 2020;202(10):1373-87. doi: 10.1164/rccm.201912-2445OC
8. Reyfman PA, Walter JM, Joshi N, et al. Single-Cell Transcriptomic Analysis of Human Lung Provides Insights into the Pathobiology of Pulmonary Fibrosis. *American journal of respiratory and critical care medicine* 2019;199(12):1517-36. doi: 10.1164/rccm.201712241OOC [published Online First: 2018/12/18]
9. Obraztsova K, Basil MC, Rue R, et al. mTORC1 activation in lung mesenchyme drives sex- and age-dependent pulmonary structure and function decline. *Nat Commun* 2020;11(1):5640. doi: 10.1038/s41467-020-18979-4 [published Online First: 2020/11/08]
10. Stuart T, Butler A, Hoffman P, et al. Comprehensive Integration of Single-Cell Data. *Cell* 2019;177(7):1888-902 e21. doi: 10.1016/j.cell.2019.05.031 [published Online First: 2019/06/11]
11. Traag VA, Waltman L, van Eck NJ. From Louvain to Leiden: guaranteeing well-connected communities. *Sci Rep* 2019;9(1):5233. doi: 10.1038/s41598-019-41695-z [published Online First: 2019/03/28]
12. Wang A, Chiou J, Poirion OB, et al. Single-cell multiomic profiling of human lungs reveals celltype-specific and age-dynamic control of SARS-CoV2 host genes. *Elife* 2020;9 doi: 10.7554/eLife.62522 [published Online First: 2020/11/10]
13. Guo M, Du Y, Gokey JJ, et al. Single cell RNA analysis identifies cellular heterogeneity and adaptive responses of the lung at birth. *Nat Commun* 2019;10(1):37. doi: 10.1038/s41467-018-07770-1 [published Online First: 2019/01/04]
14. Guo M, Morley MP, Jiang C, et al. Guided construction of single cell reference for human and mouse lung. *Nat Commun* 2023;14(1):4566. doi: 10.1038/s41467-023-40173-5 [published Online First: 20230729]
15. Chen J, Bardes EE, Aronow BJ, et al. ToppGene Suite for gene list enrichment analysis and candidate gene prioritization. *Nucleic Acids Res* 2009;37(Web Server issue):W305-11. doi: 10.1093/nar/gkp427 [published Online First: 20090522]
16. Jin S, Guerrero-Juarez CF, Zhang L, et al. Inference and analysis of cell-cell communication using CellChat. *Nat Commun* 2021;12(1):1088. doi: 10.1038/s41467-021-21246-9 [published Online First: 2021/02/19]

17. Love MI, Huber W, Anders S. Moderated estimation of fold change and dispersion for RNA-seq data with DESeq2. *Genome Biol* 2014;15(12):550. doi: 10.1186/s13059-014-0550-8
18. Kumar ME, Bogard PE, Espinoza FH, et al. Mesenchymal cells. Defining a mesenchymal progenitor niche at single-cell resolution. *Science* 2014;346(6211):1258810. doi: 10.1126/science.1258810
19. Clements D, Mayer RJ, Johnson SR. Subcellular distribution of the TSC2 gene product tuberin in human airway smooth muscle cells is driven by multiple localization sequences and is cell-cycle dependent. *Am J Physiol Lung Cell Mol Physiol* 2007;292(1):L258-66.

Uncropped scans of supplementary blots

11A

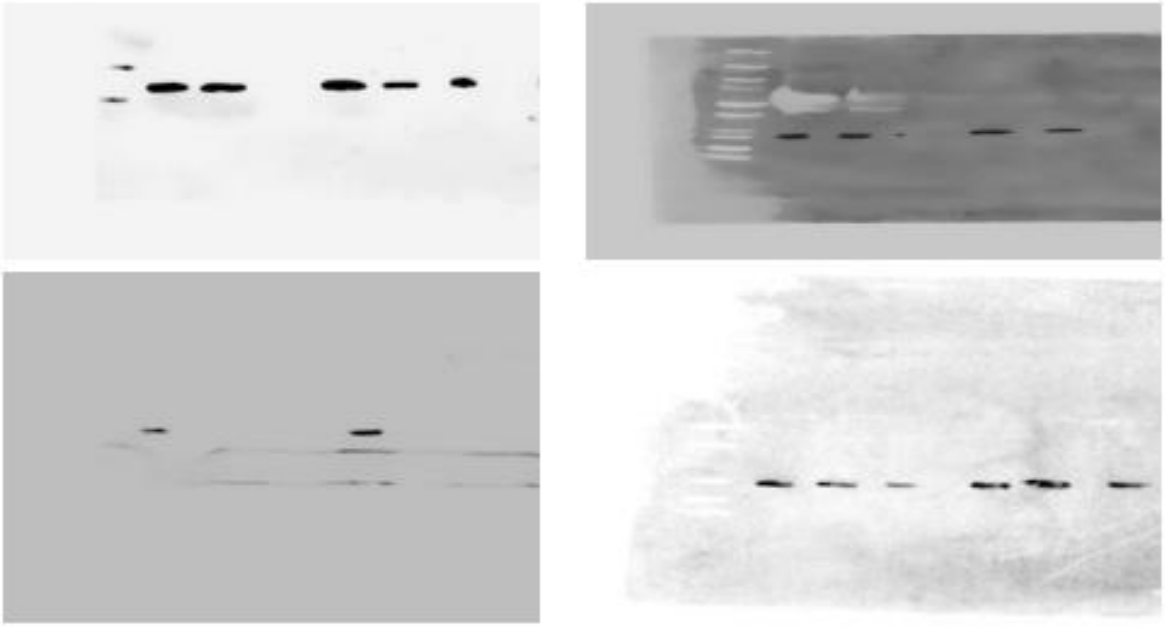

12A

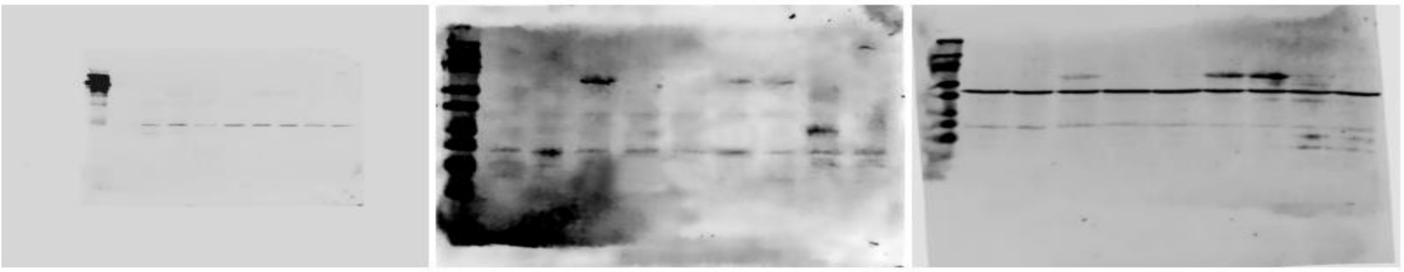

12B

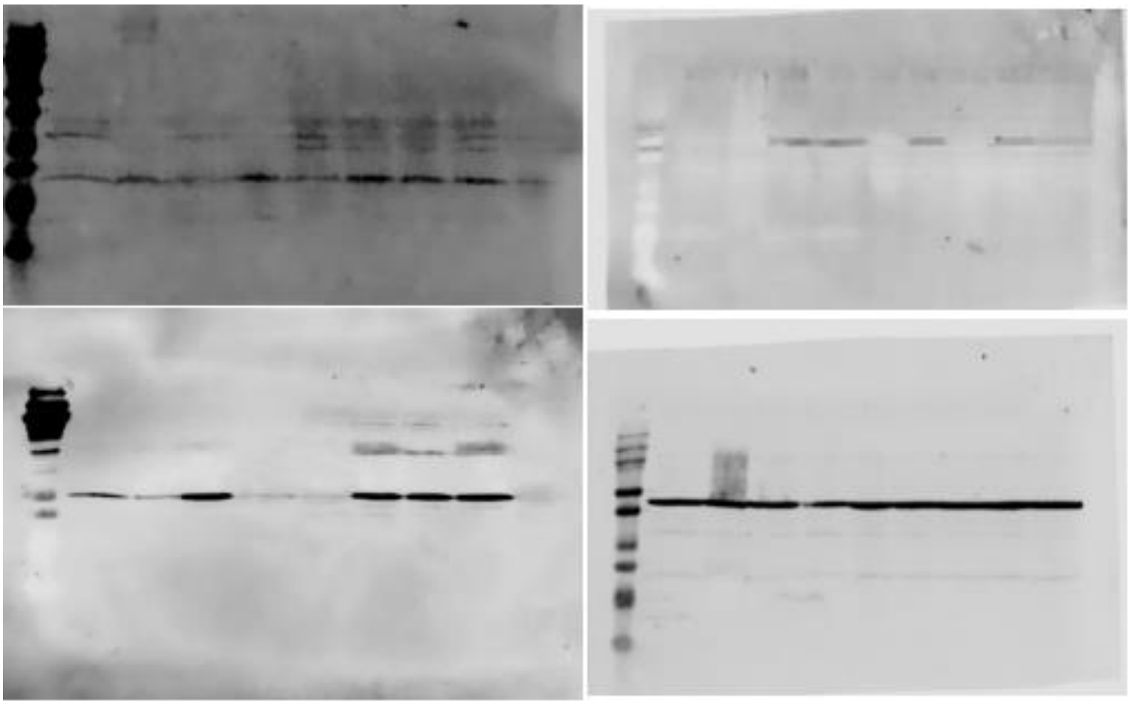

Supplement: Supplementary file 1 — Supplementary Information [file 41467_2025_64036_MOESM1_ESM.pdf]
