# Supplementary figures and images for: mTOR dysregulation induces IL-6 and paracrine AT2 cell senescence impeding lung repair in lymphangioleiomyomatosis
Source: Nat Commun. 2025 Oct 9;16:8996. doi: 10.1038/s41467-025-64036-3 (PMC12511327; doi:10.1038/s41467-025-64036-3)

p21

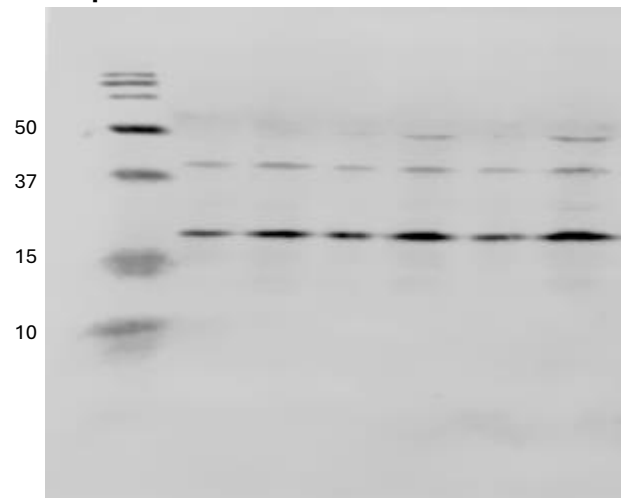

p16

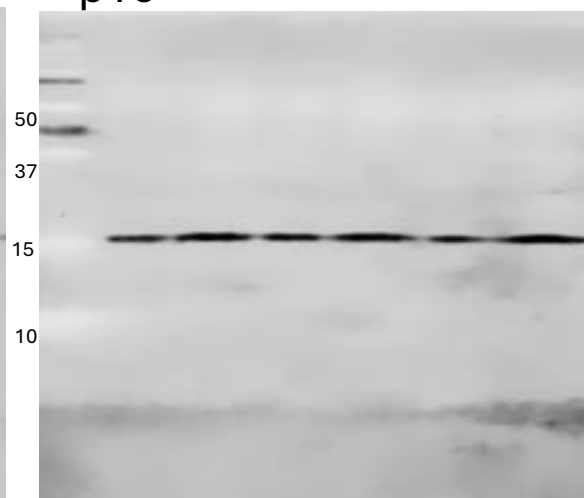

$\beta$ -actin

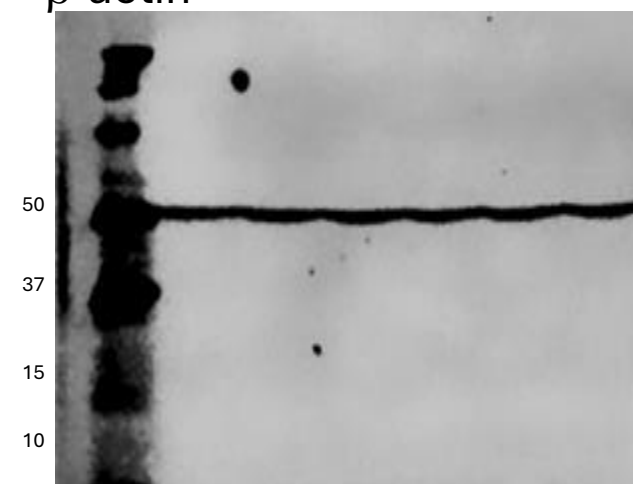

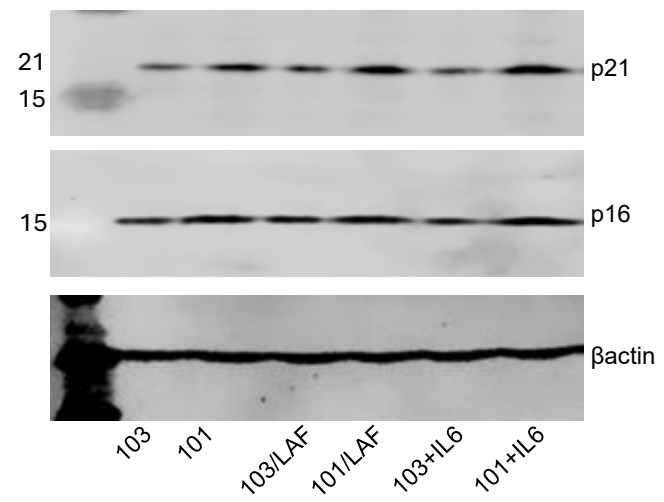

Supplement: Supplementary file 4 — Source data [file 41467_2025_64036_MOESM4_ESM.zip › 6-Bsource.pdf]
